# Effects of Self-Rated Health and Self-Rated Economic Situation on Depressed Mood Via Life Satisfaction Among Older Adults in Costa Rica

**DOI:** 10.1177/0898264315589577

**Published:** 2016-03

**Authors:** Benjamín Reyes Fernández, Luis Rosero-Bixby, Heli Koivumaa-Honkanen

**Affiliations:** 1 Universidad de Costa Rica, Costa Rica; 2 University of California, Berkeley; 3 Institute of Clinical Medicine, University of Eastern Finland, Kuopio; 4 Department of Psychiatry, Kuopio University Hospital, Finland; 5 Department of Psychiatry, South-Savonia Hospital District, Mikkeli, Finland; 6 Department of Psychiatry, North Karelia Central Hospital, Joensuu, Finland; 7 Department of Psychiatry, SOSTERI, Savonlinna, Finland; 8 Department of Psychiatry, SOTE, Iisalmi, Finland; 9 Department of Psychiatry, Lapland Hospital District, Rovaniemi, Finland; 10 Clinic of Child Psychiatry, Oulu University Hospital, Oulu, Finland

**Keywords:** depressed mood, self-rated health, life satisfaction, self-rated economic situation

## Abstract

**Objective:** The study examined the relationship of self-rated health and self-rated economic situation with depressed mood, and life satisfaction as mediator of this relationship among older adults in Costa Rica. **Method:** A longitudinal study was conducted with a subsample (*N* = 1,618) from the Costa Rican Longevity and Healthy Aging Study (CRELES). Self-rated health, self-rated economic situation, depressed mood, and life satisfaction were measured at baseline, and depressed mood was reassessed 18 months later. Putative mechanisms for changes in depressed mood were examined by means of conditional process analysis. **Results:** Self-rated health was negatively associated to depressed mood. This effect took place via life satisfaction. An interaction showed that better economic situation compensated the effect of a low self-rated health on life satisfaction. **Discussion:** This study suggests that subjective variables such as self-rated health, economic situation, and life satisfaction should be considered when addressing the onset of depressed mood.

## Introduction

Depression is not only a mental health disorder, but it is also a major public health problem affecting the population in many different ways. It is often associated to chronic medical diseases and can worsen their health outcome (Jakkula et al., 2008; Koivumaa-Honkanen et al., 2001; Soronen et al., 2008). Major depression is one of the leading causes of disease burden worldwide, and its impact is expected to grow (World Health Organization [WHO], 2008). Understanding how several risk factors antecede and play a role in the onset of depression is a key step for the formulation of adequate health policies. Special attention has to be paid to depression in aging societies like Latin America (Cotlear, 2011). Still, at the population level, depressive mood should be the focus of attention, as only a minority of depressive subjects is being diagnosed in health care (Wittchen et al., 2011).

If depression is so relevant, how can it be better understood? Which factors may contribute to understand the onset of depression? In the present study, we examine how factors such as self-rated health, self-rated economic situation, and life satisfaction have an effect on the depressed mood in older adults in Costa Rica. As pointed out by Beck and Dozois (2011), beliefs and perceptions of circumstances play a key role in the onset of depressed mood. We base our study on this approach, and thus, we focus our attention on subjective variables.

**Figure 1.**
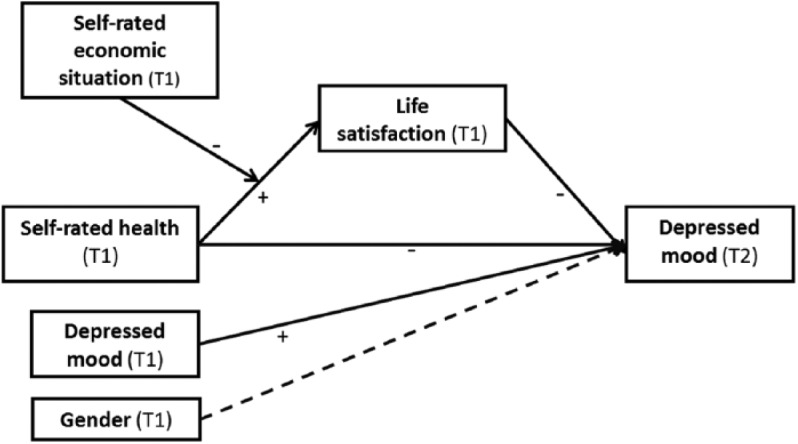
Conceptual diagram of proposed mechanism for depressed mood.

In general population, indicators of poor subjective well-being such as life dissatisfaction (Koivumaa-Honkanen et al., 2000, 2001; Koivumaa-Honkanen, Kaprio, Honkanen, Viinamaki, & Koskenvuo, 2004; Koivumaa-Honkanen et al., 2012; Paunio et al., 2009) or poor self-rated health (Cole & Dendukuri, 2003) can reveal those with risk of depression and its adverse consequences. There is also evidence showing a relationship between poor self-rated economic situation and depressed mood (Bridges & Disney, 2010). In the present study, we examined the relationship of self-rated health and self-rated economic situation with depression, and life satisfaction as mediator of this relationship among older adults in Costa Rica (Figure 1).

### Self-Rated Health and Depressed Mood

The relationship between self-rated health and depressed mood is of negative nature, as decrements in health status, which comes with aging and risky lifestyles, are related to increased depressive symptoms and increased risk of depression (Ayuso-Mateos, Nuevo, Verdes, Naidoo, & Chatterji, 2010; French, Sargent-Cox, & Luszcz, 2012; Viinamaki et al., 2008). Perception of one’s own health status is a simple, practical way of portraying actual health status (Jylhä, 2009; Jylhä, Volpato, & Guralnik, 2006; Quesnel-Vallée, 2007). Perceived poor health or illness is a known predictor of depression episodes and depressive symptoms (Stefansson et al., 2008; Viinamaki et al., 2008). However, this relationship needs further investigation, as depressed mood is the outcome of adverse processes, not a direct result of objective conditions. Thus, perception of illness and poor health does not necessarily and directly lead to depressed mood (Brandthill et al., 2001; Rothermund & Brandtstädter, 2003). For example, physically ill persons with self-confidence and internal locus of control may actively cope with the adverse situation and, thus, be protected against depressed mood (Stewart & Yuen, 2011).

One possible mediating factor between poor self-rated health and subsequent depressed mood is life satisfaction, as decreasing health might first decrease satisfaction with life and later increase depressive symptoms. Thus far, both life satisfaction (Koivumaa-Honkanen et al., 2004) and good self-rated health (French et al., 2012) have been reported to be negatively related to depressed mood, but good self-reported health has also predicted life satisfaction (Putzke, Richards, Hicken, & DeVivo, 2002; Rissanen et al., 2011). Thus, it is worthwhile to study whether life satisfaction might work as a mediator in the negative relationship between self-rated health and depressed mood (Figure 1).

It can be argued that a life satisfaction and depressed mood are two opposite faces of the same coin, and therefore, the role of life satisfaction as explanation of the onset of depressed mood is spurious. However, there are clear distinctions between them. Although life satisfaction measures aspects of both good and poor mental health, depressive symptoms deal only with the existence or non-existence of adverse mental symptoms. Thus, a person unsatisfied with life is not necessarily a depressed person and vice versa, and an intervention aimed to treat depression might reduce depressed mood but that does not mean the person will become satisfied with life at the same time (Koivumaa-Honkanen et al., 2008).

### Self-Rated Economic Situation and Life Satisfaction

Economic circumstances can be important for determining life satisfaction and mood not only because of the objective situation but also because of the person’s subjective interpretation of it (Beck & Dozois, 2011). An individual can value his or her economic situation depending on a social context, the situation of others, or the own past living standards. Evidence has shown that individuals differ in their psychological response to objective financial situations and that the subjective dimension has stronger associations to mood and reported depression (Bridges & Disney, 2010; Hayo & Seifert, 2003; Senik, 2009). Our causal framework thus includes subjective economic situation instead of objective variables of income or wealth.

Previous literature has found direct effects of economic situation on life satisfaction, but the contribution of the interaction of self-rated economic situation with other variables such as health perception has not been studied (Lue, Chen, & Wu, 2010). Our framework posits that when individuals experience deteriorations in their health, this could be compensated by the perception of available economic resources. In other words, the relationship between poor self-rated health and life dissatisfaction might be more pronounced among those who experience their economic situation as worse, as they have no possibility to look for the benefits that economic sources can bring, for example, in adapting to or getting treatment for their disease. Thus, one could suggest that the self-rated economic situation might be a moderator of the relationship between health perception and life satisfaction as shown in Figure 1.

### Aims and Hypotheses

The study examined the relationship of self-rated health and self-rated economic situation with depressed mood, and life satisfaction as mediator of this relationship among older adults in Costa Rica. Three hypotheses were as follows:

**Hypothesis 1:** Self-rated health is negatively associated to depressed mood.
**Hypothesis 2:** The negative association between self-rated health and depressed mood can be partially explained via life satisfaction.
**Hypothesis 3:** The effect of self-rated health on life satisfaction is less pronounced among those with higher subjective economic situation, because good economic situation can compensate the impact of a poor health on life satisfaction.

If the hypotheses are supported by our data, this knowledge on the factors that may lead to a depressed mood can be used in situations when preventive measures are needed.

## Method

### Participants and Procedure

Data were taken from the Costa Rican Longevity and Healthy Aging Study (CRELES). CRELES is a nationally representative longitudinal study, currently with three waves, measuring many different dimensions of the aging process among Costa Ricans, from biomarkers to social and psychological variables (Gersten, Dow, & Rosero-Bixby, 2010; Rosero-Bixby, Fernández, & Dow, 2013). The CRELES sample was drawn from Costa Rican residents in the 2000 population census who were born in 1945 or before with oversampling of older ages. The first wave (T1) of interviews took place mainly during 2005, so the age of participants is 60 years or higher at baseline. Wave 2 (T2) took place mainly in 2007. The ethics committee of the University of Costa Rica approved the study, and all participants granted their informed consent by means of their signature. Interviews and specimen collection took place in the homes of participants.

From the original sample at baseline (*N* = 2,827), 954 older adults were not taken into account for the subjective variables due to cognitive impairment, screened by means of mini-mental at T1 and T2. Moreover, there was some dropout from T1 to T2 due to death (*n* = 101). Finally, there were an additional loss of 154 subjects at T2, that was not due to death or cognitive impairment, and there were 1,618 completers. There was less than 0.05% of missing values for each of the study variables. Thus, the longitudinal sample for analysis was *N* = 1,608.

### Measures

Except for the gender variable (man = 1, woman = 2), in all variables in the analysis, coding was done so that greater intensity of the measured properties is reflected by higher numbers.

*Depressed mood* was measured both at T1 and T2 using the 15-item Geriatric Depression Scale (GDS-15; Sheikh & Yesavage, 1986; Table 1). GDS-15 is a shortened version of the original 30 items (Yesavage et al., 1982). Reviews show its criterion validity for screening purposes (Wancata, Alexandrowicz, Marquart, Weiss, & Friedrich, 2006). Psychometric properties have been reported for its Spanish long and short versions (Fernández-San Martín et al., 2002; Ortega Orcos, Salinero Fort, Kazemzadeh Khajoui, Vidal Aparicio, & de Dios del Valle, 2007).

**Table 1.** The Geriatric Depression Scale (GDS-15): 15 Items and Scoring Rules.

Choose the best answer for how you have felt over the past week:	
1. Are you basically satisfied with your life?	YES/**NO**
2. Have you dropped many of your activities and interests?	**YES/**NO
3. Do you feel that your life is empty?	**YES/**NO
4. Do you often get bored?	**YES/**NO
5. Are you in good spirits most of the time?	YES/**NO**
6. Are you afraid that something bad is going to happen to you?	**YES/**NO
7. Do you feel happy most of the time?	YES/**NO**
8. Do you often feel helpless?	**YES/**NO
9. Do you prefer to stay at home, rather than going out and doing new things?	**YES/**NO
10. Do you feel you have more problems with memory than most?	**YES/**NO
11. Do you think it is wonderful to be alive now?	YES/**NO**
12. Do you feel pretty worthless the way you are now?	**YES/**NO
13. Do you feel full of energy?	YES/**NO**
14. Do you feel that your situation is hopeless?	**YES/**NO
15. Do you think that most people are better off than you are?	**YES**/NO

*Note*. Answers in bold indicate depression. Score 1 point for each bolded answer. A score > 5 points = suggestive of depression.

In accordance to the scoring rules of the GDS-15, an item indicating presence of depressed mood (bolded responses in Table 1) was scored as 1, and an item indicating absence of depressed mood was scored as 0. Thus, the final sum score ranged between 0 and 15, higher scores representing higher depressed mood. GDS-15 is a screening method, with a score >5 indicating probable depression (D’Ath, Katona, Mullan, Evans, & Katona, 1994). The Cronbach’s alpha for these 15 items was 0.85, indicating high internal validity of the scale. The score of this variable was later on normalized (0-1) for interpretation purposes.

*Self-rated health* was measured at T1 using the item “How would you say is your health now?” with five response alternatives, from poor (5), fair (4), good (3), very good (2), to excellent (1). This item was reversed and normalized; thus, poor = 0 and excellent = 1, following the scoring rules of Ware and Gandek (1998). This is a usual format and wording to measure, although there is no uniformity in the item formulation and response options (Idler & Benyamini, 1997). Even though it consists of one single item, its relationship with mortality, considered the gold standard of objective measures of health (Quesnel-Vallée, 2007), can be taken as strong evidence of its validity.

*Life satisfaction* was measured at T1 with the item “In general, how do you feel about your life?” with a 4-point response format. The response options were as follows: very dissatisfied (4), somewhat dissatisfied (3), somewhat satisfied (2), and very satisfied (1). We reversed and transformed it into a 0 to 1 scale, with 0.33 increments; thus, very dissatisfied = 0 and very satisfied = 1.

*Self-rated economic situation* was obtained at T1 by interviewees by the item “How would you describe your current economic situation?” with a 5-point response format: excellent (1), very good (2), good (3), fair (i.e., regular = 4), and bad (5). Again, we reversed and transformed it into a 0 to 1 scale with 0.25 increments; thus, bad = 0 and excellent = 1.

### Data Analysis

Attrition analyses were conducted with MANOVA for continuous variables and χ^2^ for categorical variables.

**Figure 2.**
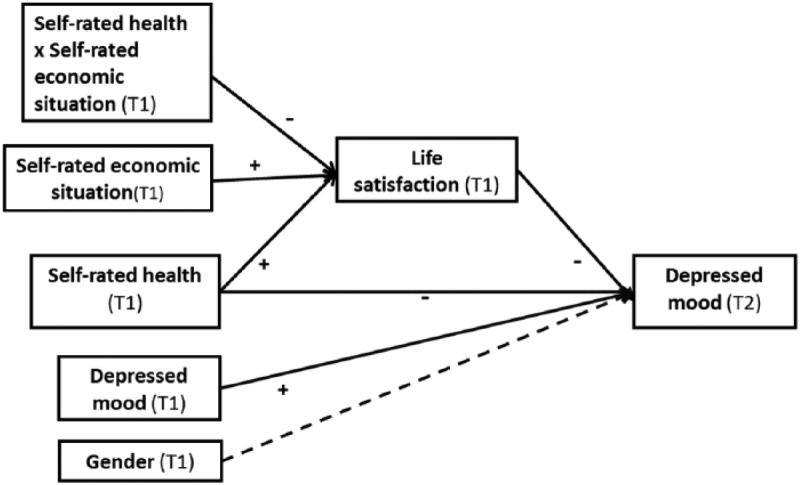
Statistical diagram of the proposed mechanism for depressed mood.

To test the hypothesis proposed, conditional process analyses were carried out, namely, mediated moderation (Hayes, 2013). Mediation describes how an independent variable affects a dependent variable via a third variable (the mediator). Moderation mechanisms reveal among whom this relationship works. A mediator might emerge in one group (e.g., affluent persons) but not in another (e.g., less affluent persons). The group variable operates as a moderator of the mediating relationship. In the present study, self-rated health was used as a predictor of T2 depressed mood (direct effect) and life satisfaction as a putative mediator that might translate this prediction into T2 depressed mood (indirect effect). Self-rated economic situation was specified as moderator; it means the interaction between self-rated health and self-rated economic situation is expected to have an effect on life satisfaction besides their direct effects. T1 depressed mood and gender were used as covariates on T2 depressed mood. Figure 2 shows the statistical diagram for the proposed mechanism. To probe the interaction between depressed mood and self-rated health, the Johnson–Neyman technique was used (Hayes, 2013; Johnson & Neyman, 1936). Analyses were carried out using the SPSS macro PROCESS (Hayes, 2013). Estimates of all paths were calculated by ordinary least squares procedures. In moderated mediation models, as the one examined in this study, the use of bootstrapped confidence intervals (CI) is recommended, as they need no assumption about the normality of the distribution and provide a more sounding region of significance for the indirect effect found (Preacher, Rucker, & Hayes, 2007). The number of bootstrapped resamples was 5,000, yielding 95% CI of the parameter estimates.

## Results

### Attrition Tests

When studying differences between completers (*N*_comp_ = 1,618) and non-completers (*n*_non_ = 154), no differences were found for baseline depressed mood, *M* (*SD*)_comp_ = 0.19 (0.21) versus *M* (*SD*)_non_ = 0.20 (0.21), *ns*; life satisfaction, *M* (*SD*)_comp_ = 0.90 (0.19) versus *M* (*SD*)_non_ = 0.91 (0.18), *ns*; self-rated economic situation, *M* (*SD*)_comp_ = 0.36 (0.23) versus *M* (*SD*)_non_ = 0.37 (0.23), *ns*; self-rated health, *M* (*SD*)_comp_ = 0.44 (0.25) versus *M* (*SD*)_non_ = 0.46 (0.26), *ns*; age, *M* (*SD*)_comp_ = 72 (7.72) versus *M* (*SD*)_non_ = 73 (7.48), *ns*; and gender, women_comp_ = 54% versus women_non_ = 50%, *ns*.

### Descriptive Results

The mean (*SD*) age of the sample was 71.2 (7.22) years (range = 60-93 years), 55% being women. Altogether, 70.6% had an elementary educational level, 12.3% had no education at all, while 9.5% reported a secondary level and 7.5% a para-university or university level. Only 1% of participants were very unsatisfied with life, and 5.4% considered their health as poor. Economic situation was assessed as bad by 12.8% of the sample; 16.1% had probable depression (GDS-15 > 5).

Both self-rated health at T1 and life satisfaction at T1 were negatively associated with both T1 and T2 depressed mood. T1 depressed mood was positively associated with T2 depressed mood. T1 self-rated economic situation was positively associated with T1 life satisfaction and negatively with both T1 and T2 depressed mood. The means, standard deviations, and intercorrelations of all variables used in the moderated mediation model can be seen in Table 2. No correlation was >.90, and all the Variance Inflation Factors were away from 10. Thus, major concerns on multicollinearity were dismissed (Stevens, 2009).

**Table 2.** Means (*SD*) and Intercorrelations for Self-Rated Health, Self-Rated Economic Situation, Life Satisfaction, Depressed Mood (T1 and T2), and Gender.

Variables	1	2	3	4	5	6
*M* (*SD*)	0.44 (0.25)	0.36 (0.23)	0.90 (0.19)	0.19 (0.21)	0.18 (0.21)	1.54 (0.50)
1. Self-rated health (T1)	1					
2. Self-rated economic situation (T1)	.33***	1				
3. Life satisfaction (T1)	.28***	.23***	1			
4. Depressed mood (T1)	−.36***	−.25***	−.56***	1		
5. Depressed mood (T2)	−.29***	−.18***	−.42***	.54***	1	
6. Gender	−.07**	.05*	−.04	.11***	.09**	1

*Note.* Continuous variables are normalized (rank = 0-1). Gender: men = 1, women = 2.

* Correlation is significant at the .05 level (two-tailed). **Correlation is significant at the .01. ***Correlation is significant at the .001 level (two-tailed).

### Mediated Moderation Analysis

**Figure 3.**
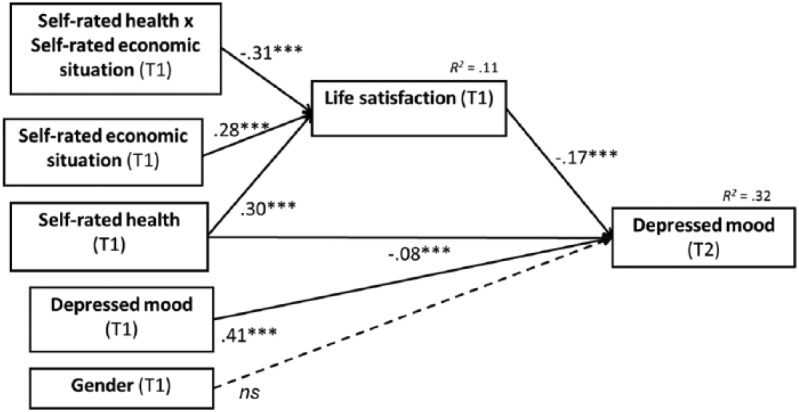
Results of the mediated moderation model for depressed mood (*N* = 1,608). *Note.* Gender: men = 1, women = 2. Variables normalized to scales 0 to 1. ***p < .001.

The main results are shown in Figure 3. The coefficients show effects between variables measured in scales from 0 to 1. For example, going from 0 to 1 in the scale of self-rated economic situation results in an increase of 0.28 in the scale 0 to 1 life satisfaction. As a first step, life satisfaction (T1) was regressed on self-rated health (T1), self-rated economic situation (T1), and their interaction. Life satisfaction was predicted by all of them. In a second step, depressed mood (T2) was regressed on self-rated health (T1), life satisfaction (T1), the covariates baseline depressed mood (T1), and gender. The indirect effect of self-rated health on depressed mood through life satisfaction was found to fall within the bootstrapped CI, 95% at +1 *SD* (*b* = −.01, CI [−0.03, −0.01]), at the mean (*b* = −.03, CI [−0.05, −0.02]), and at −1 *SD* (*b* = −.05, CI [−0.07, −0.02]) of self-rated economic situation. After controlling for life satisfaction, the direct negative relationship between self-rated health and depressed mood was still within the CI (*b* = −.08, 95% CI [−0.11, −0.04]). Put it differently, when self-rated health decreases, depressed mood increases directly and via life satisfaction as a mediator variable.

The ratio of indirect to direct effect when self-rated economic situation is at the mean is .37, but it increases to .62 when self-rated economic situation is −1 *SD*. Thus, most of the total effect of a bad self-rated health on depressed mood seems to take place directly, but the proportion of the effect that takes place via life satisfaction increases as the economic situation is perceived as worse.

**Figure 4.**
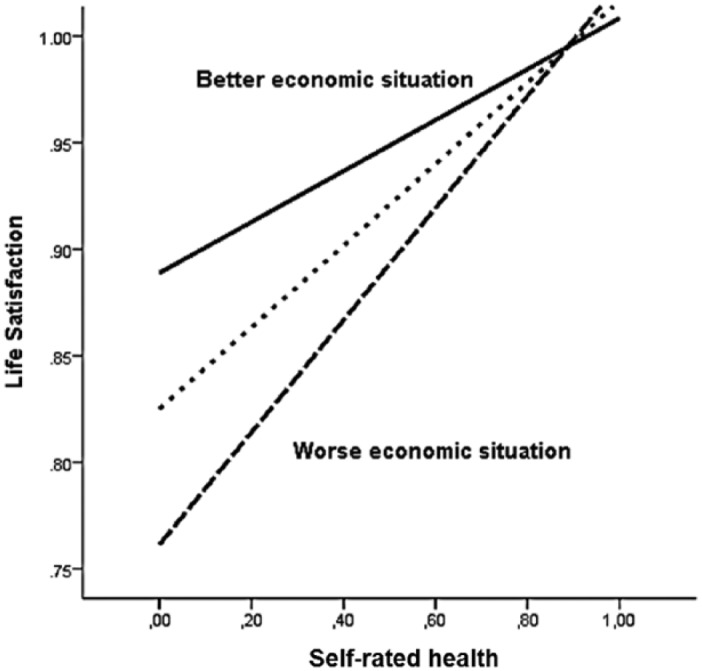
Moderation of self-rated health and self-rated economic situation on life satisfaction. *Note.* The regression lines display the effect of self-rated health on life satisfaction on the level of the mean, 1 *SD* above and 1 *SD* below the mean of self-rated economic situation. Variables normalized to scales 0 to 1.

The moderator effect of self-rated economic situation between self-rated health and life satisfaction is depicted in Figure 4. For those who were less affluent, a deteriorated self-rated health was more negatively associated with life satisfaction and, thereby, with depressed mood. However, the effect of deteriorated health on life satisfaction disappears at higher levels of perceived economic situation (data not shown). As shown by the Johnson–Neyman technique, this effect was no longer significant when self-rated economic situation was *b* ≥ .80 (CI [−0.01, 0.12]), which means that for the most affluent older adults, perceived health was not a predictor of satisfaction.

In the model, gender and T1 depressed mood were used as covariates of T2 depressed mood. Gender played no significant role in the model to predict depressed mood (Figure 3). However, T1 depressed mood had a significant contribution in the model.

## Discussion

Our study in older adults in Costa Rica indicated that subjects with good self-rated health regardless of gender were less likely to suffer from depressed mood. If this person’s self-rated health decreases, the probability to suffer afterward from depressed mood increases. This effect is partially explained by the impact of self-rated health on life satisfaction. However, a decreased self-rated health does not necessarily lead to a decrease in life satisfaction. The negative impact of poor self-rated health on life satisfaction can be compensated by a good self-rated economic situation. Put it differently, self-rated economic situation is a moderator of the relationship between self-rated health and life satisfaction. Thus, the onset of depressed mood may be the product of a mechanism involving self-rated health, self-rated economic situation, and life satisfaction.

The negative association found between self-rated health and depressed mood is consistent with previous literature (Ayuso-Mateos et al., 2010; Dickens et al., 2008). Nevertheless, it was found in the present study that this effect can be partially explained by life satisfaction. Decreased self-rated health can lead to life dissatisfaction, which consequently increases depressed mood. It was also revealed that the path from low self-rated health to life dissatisfaction seems to be different for those with high and low self-rated economic situation.

Thus, according to the present study, if someone has poor self-rated economic situation, the perception of having a bad health is associated to life dissatisfaction, but if he or she has a good economic situation, the effect of poor self-rated health on life satisfaction is weak and non-significant. Put differently, a perceived good economic situation compensates the effects of a perception of bad health.

This finding concurred with those of Loewe, Bagherzadeh, Araya-Castillo, Thieme, and Batista-Foguet (2014) in the Chilean context, in that satisfaction with health and satisfaction with finances contributed significantly to life satisfaction, although they did not include any interaction analysis. Our results are also parallel to those reported by Rojas (2009). Based on a cross-sectional general sample of Costa Ricans, he concludes that economy may compensate the impact of health limitations on subjective well-being. Our results are also very similar to those reported by Lue et al. (2010) from a study conducted with older adults in Taiwan. They found that women who perceived health and financial stress, who were less satisfied with life, and who had worse functional condition were more likely to suffer from depression. However, they did not try to disentangle any mediation or moderation mechanism to understand how the onset of depression takes place.

The present study offers longitudinal evidence showing that low life satisfaction works as a step toward depressed mood in a psychological process where health, when perceived as negative, and where economic resources, when perceived as unavailable, lead to dissatisfaction and further to depressed mood. Thus, subjective variable factors such as self-rated health, self-rated economic situation, and life satisfaction should be taken into account in health screening and health policies. When life satisfaction decreases, it should be considered as a warning to intervene and to prevent depressed mood. Life satisfaction is briefly assessed, valid, and informative, no matter whether interventions are implemented either for persons already depressed or for persons not yet depressed. However, people who suffer from a disadvantageous economic situation and health problems deserve particular attention. Some limitations and comments are to be mentioned. Diagnostic procedures for depression were not used, as it is expensive and difficult to apply to such large number of participants, but a valid and well-known measure for screening depressed mood among the elderly was used. Thus, it was possible to study the aims of the present study. The relationship found between self-rated health, self-rated economic situation, and life satisfaction might be to some extent specific for Costa Rican population. In different contexts, the role of economic and social factors may vary (Dolan, Peasgood, & White, 2008), maybe because of cultural differences or because of the specific organization of these societies. Single-items were used for the measurement of several variables, what might arise some concerns on reliability. Still, single-item self-rated health and self-rated economic situation are broadly used. They were measured at the ordinal level. The subjective nature of the variables measured could also be criticized. However, as pointed out by Beck and Dozois (2011), the interpretation of circumstances and, thus, subjective variables, play a key role to understand depressed mood. Objective measures might not be sensitive to contextual factors and one’s own past living standards, which among other subjective factors are probably more important when determining the effect of wealth on well-being and mood (Hayo & Seifert, 2003; Senik, 2009).

Thus, the strengths of the present study are its focus on a psychological mechanism behind depressed mood with a national representative study. Previous studies have focused on the effect of objective variables on health perception (Brenes-Camacho, 2011), but we concentrated on subjective variables and possible psychological mechanism leading to depressed mood or life satisfaction among Costa Rican older adults. This knowledge is particularly relevant in the context of demographic aging in Latin America (Cotlear, 2011).

Our findings suggest an option to prevent depressed mood, namely, improving life satisfaction. Poor health and disability are prone to become more common at advanced ages (Romero-Ortuno & Kenny, 2012), and both of them can be associated with poor self-rated health (Mavaddat, Van der Linde, Savva, Brayne, & Mant, 2013). However, depressed mood is not their necessary and inevitable consequence (Rothermund & Brandtstädter, 2003). There is a psychological process in between which involves life satisfaction, which is one of the main components of subjective well-being (Vaillant, 2003). Thus, the mediation role of life satisfaction in the onset of depressed mood emphasizes the preventive role of subjective well-being. There is need for intervention, strategies, and research that focus on improving and preserving subjective well-being among the elderly. These could include not only improving one’s social situation and social support but also improving health behavior (Koivumaa-Honkanen et al., 2012; Rissanen et al., 2011), other behavioral activation, and overall coping strategies (Honkalampi et al., 2001; Koivumaa-Honkanen et al., 2001), which will enable a subject to face adversities in health and in life. The role of coping strategies to successfully deal with health and economic difficulties, and to lead to well-being and resiliency, is supported by the literature (Gaston-Johansson et al., 2013; Hobfoll, 2011; Wadsworth, 2012). The relevance of such interventions seems to be higher among non-wealthy older adults, as the impact of a deteriorated health on their mood is higher. Also the possibility of implementing measures to improve their self-rated economic situation should be taken into account by policy makers.
